# Cluster Formation Effect of Water on Pristine and Defective MoS_2_ Monolayers

**DOI:** 10.3390/nano13020229

**Published:** 2023-01-04

**Authors:** Kangli Wang, Beate Paulus

**Affiliations:** Institut für Chemie und Biochemie, Freie Universität Berlin, Arnimallee 22, 14195 Berlin, Germany

**Keywords:** water cluster, molybdenum disulfide, optical properties, Bethe–Salpeter equation

## Abstract

The structure and electronic properties of the molybdenum disulfide (MoS_2_) monolayer upon water cluster adsorption are studied using density functional theory and the optical properties are further analyzed with the Bethe–Salpeter equation (BSE). Our results reveal that the water clusters are electron acceptors, and the acceptor tendency tends to increase with the size of the water cluster. The electronic band gap of both pristine and defective MoS_2_ is rather insensitive to water cluster adsorbates, as all the clusters are weakly bound to the MoS_2_ surface. However, our calculations on the BSE level show that the adsorption of the water cluster can dramatically redshift the optical absorption for both pristine and defective MoS_2_ monolayers. The binding energy of the excitons of MoS_2_ is greatly enhanced with the increasing size of the water cluster and finally converges to a value of approximately 1.16 eV and 1.09 eV for the pristine and defective MoS_2_ monolayers, respectively. This illustrates that the presence of the water cluster could localize the excitons of MoS_2_, thereby greatly enhance the excitonic binding energy.

## 1. Introduction

Recently, layered transition metal dichalcogenides (TMDs) have gained great attention due to their unusual electronic and optical properties, including sizable band gap, strong photoluminescence, excitonic effect, valley-specific circular dichroism, and so on [1,2,3,4,5,6,7,8]. For instance, while the bulk MoS_2_ is an indirect band gap semiconductor, monolayer MoS_2_ is a direct band gap semiconductor. Importantly, the exciton in the MoS_2_ monolayer is strongly bound because of the reduced dimensionality. The presence of intrinsic structural defects in TMDs [9,10,11], which is inevitable in the experiments, provides even more possibility to tune the reactivity and transport properties and create new functionalities. For example, NO molecules can chemically adsorb on the sulfur (S) defects of the MoS_2_ monolayer [12,13,14], indicating the high sensitivity towards NO molecules and the great potential of TMDs as gas sensors. These suggest that the layered TMDs, in particular MoS_2_, are promising candidates for graphene replacement with various application possibilities.

In reality, the humidity, i.e., the effect of adsorption of water, plays an important role in countless applications and technological processes, since water is present in the environment of almost any device. Examples include field-effect transistors [15], gas sensors [16,17], and electronic devices [18]. In addition, various water clusters (H_2_O)*_n_* can form via intermolecular hydrogen bonds on the surface of a solid depending on the environmental conditions [19,20,21,22,23,24,25,26]. Therefore, the understanding of the properties of water/solid interface is very important for the development and improvement of various applications.

The great potential of the MoS_2_ monolayer and the importance of the water/solid interface call for a theoretical perspective on how the presence of water influences the electronic and optical properties of MoS_2_. A single water molecule was theoretically predicted to only weakly adsorb on the MoS_2_ surface compared with other inorganic molecules, and had no influence on the electronic band gap [12,13,27,28,29,30]. However, there is still a lack of theoretical understanding of the influence of a single water molecule on the optical and excitonic properties. More importantly, the effect of water clusters associated with hydrogen bonds on the electronic and optical properties of the MoS_2_ monolayer is still largely unexplored.

It is well known that the GW approximation combined with the Bethe–Salpeter equation based on DFT (DFT-GW-BSE) [31,32,33] can accurately model photoemission measurements and optical absorption. In this work, by employing this method, we analyze how the water cluster influences the electronic, optical and excitonic properties of both pristine and defective MoS_2_ monolayers. In this model, a water cluster consisting of up to five water molecules is considered to adsorb on a large supercell of the MoS_2_ monolayer.

## 2. Computational Details

All calculations except for the charge density difference are performed using GPAW code [34] based on the projector augmented wave method. To describe the exchange and correlation effects, including the dispersion interaction, we adopt the vdW-DF-CX functional [35] to obtain the structures upon water cluster adsorption. A cutoff energy of 500 eV for the plane-wave basis set and a Monkhorst–Pack k-point sampling of 6 × 6 × 1 are employed. The S defect is the main factor among the intrinsic structural defects in TMDs. Therefore, in order to ensure that the S defect density in our simulation models is in the same magnitude of experimental density (10^13^ cm^−2^) [9,36], one S defect is created in a (4 × 4) supercell of the MoS_2_ monolayer, corresponding to a defect density of 6.2 × 10^13^ cm^−2^. A vacuum space of more than 15 Å is chosen during geometry optimization to avoid undesired interactions between neighboring supercells in the perpendicular direction. The clusters containing up to five water molecules are considered to absorb on the MoS_2_ surface. All the structures are fully relaxed until the maximum force acting on each atom is less than 0.02 eV/Å and the energy change is less than 10^−5^ eV. Within this frame, we obtain a lattice constant of 3.18 Å and a Mo-S bond length of 2.41 Å for the pristine MoS_2_, which are in good agreement with experimental results [37] and previous simulations [38,39]. Using the optimized structures, the charge transfer between adsorbate and substrate is discussed by means of Bader analysis [40]. The charge density calculation is performed by means of the DFT method at the vdW-DF-CX level using the plane wave Vienna ab initio Simulation Package (VASP) code [41,42] and the same computational parameters as for the GPAW calculations.

**Figure 1:**
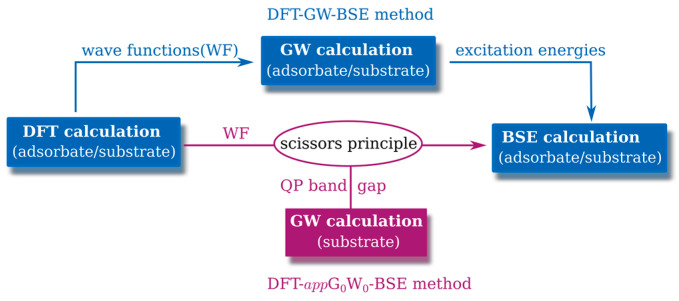
Schematic illustration of DFT-GW-BSE [31,32,33] and DFT-*app*G_0_W0-BSE.

**Figure 2:**
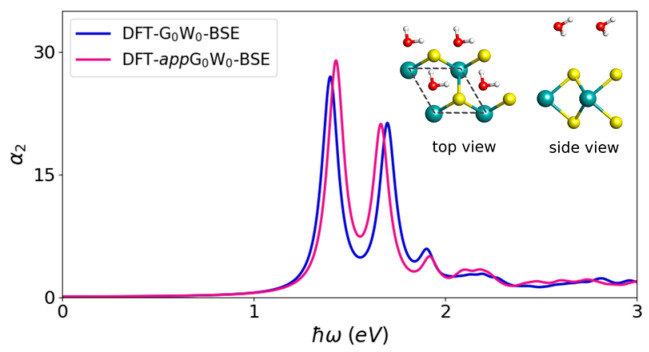
Optical absorption spectra for MoS_2_ with one H_2_O molecule in a unit cell by DFT-G_0_W_0_-BSE and DFT-*app*G_0_W_0_-BSE methods with PBE functional. The spectra are obtained by applying a Lorentzian broadening of 0.05 eV. For both cases, the electronic band gap is 2.48 eV. The corresponding structure is also shown.

The DFT-GW-BSE method is a three-step procedure to determine the electronic and optical properties of a solid. The first step is to obtain the Kohn–Sham energies and wave functions by DFT calculation. In the second step, the quasi-particle (QP) band structure energies are obtained by GW approximation. Finally, the BSE is solved to get the coupled electron–hole excitation energies and exciton wave functions. Motivated by the weak interaction between adsorbate and substrate and the high cost of GW calculation, we use an approximated method, DFT-*app*G_0_W_0_-BSE (one-shot G_0_W_0_: GW equations are not solved self-consistently), to perform the BSE calculation. In this method, the electron–hole interaction is approximated by applying the scissor operator to the DFT band structure of the adsorbate/substrate system according to the QP band gap of the substrate (as displayed in Figure 1), motivated by the negligible influence of the adsorbate on the electron–hole interaction in a certain range. To enable a more direct comparison with DFT-GW-BSE, we apply the DFT-G_0_W_0_-BSE and DFT-*app*G_0_W_0_-BSE methods to the case of a single H_2_O molecule on the pristine MoS_2_ unit cell. The corresponding results are displayed in Figure 2. It is clear that the optical absorption spectrum calculated by the DFT-*app*G_0_W_0_-BSE method is in good agreement with that by the DFT-G_0_W_0_-BSE method within 0.05 eV deviation.

After successfully applying the DFT-*app*G_0_W_0_-BSE method to describe the optical properties of a single H_2_O molecule on the pristine MoS_2_ unit cell, we extend our investigation to the optical properties of the water cluster on a (4 × 4) supercell. The DFT calculation is performed by the general gradient approximation along with the Perdew–Burke–Ernzerhof (PBE) exchange–correlation functional [43]. Our previous study [39] shows that for a (4 × 4) supercell, 10 Å vacuum with 50 eV G_0_W_0_ self-energy and 3 × 3 × 1 k-point sampling is sufficient to obtain the converged QP band gap by employing the 2D truncation. Therefore, the same parameters are applied to the G_0_W_0_ calculation of the pristine and defective MoS_2_. To obtain converged optical spectra, the 16 highest valence bands and the 32 lowest conduction bands are used as a basis to calculate excitonic contribution with a large k-point grid of 8 × 8 × 1. The spectrum is obtained by applying a Lorentzian broadening with a fullwidth half maximum of 0.05 eV. 

## 3. Results

### 3.1. Configurations of (H_2_O)_n_ on MoS_2_ Monolayers

**Figure 3:**
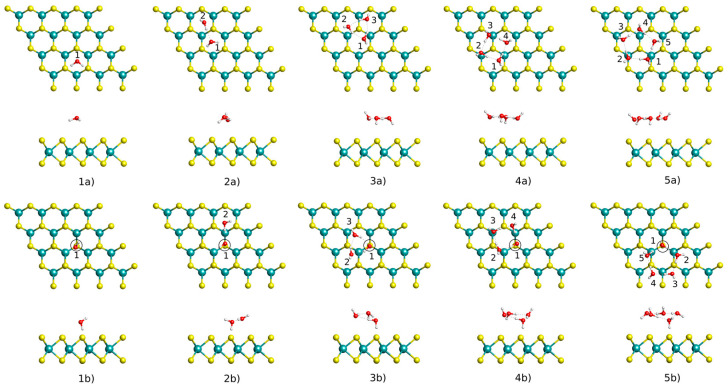
The most favorable configurations for (H_2_O)*_n_* (*n* = 1–5) on the pristine and defective MoS_2_ monolayers obtained by geometry optimization with the vdW-DF-CX functional. The notations of (**1a**–**5a**) and (**1b**–**5b**) correspond to the pristine and defective surface, respectively; numbers 1–5 correspond to the number of water molecules on the surface. The S defect is represented by the black circle.

We first investigate the adsorption of (H_2_O)*_n_* on both pristine and defective MoS_2_ surfaces by identifying the most stable adsorption structures. Various high-symmetry adsorption sites and adsorption orientations are considered; the most favorable configurations and corresponding results are presented in Figure 3 and Table 1, respectively. We discriminate between the adsorption energy of the whole cluster on MoS_2_ and the binding energy, which is referred to the isolated water molecules. The adsorption energy is calculated as

(1) $$E_{ad}=E_{tot}-E_{{MoS}_{2}}-E_{cluster}$$

where $E_{tot}$ represents the total energy of MoS_2_ with cluster adsorbed, $E_{{MoS}_{2}}$ and $E_{cluster}$ are the energies of the MoS_2_ monolayer and the isolated cluster. The binding energy is determined using

(2) $$E_{bind}=E_{tot}-E_{{MoS}_{2}}-{nE}_{H_{2}O}$$

where $E_{tot}$, $E_{{MoS}_{2}}$, $E_{H_{2}O}$, and *n* are the total energy of MoS_2_ with the cluster adsorbed, the energy of the MoS_2_ monolayer, the energy of relaxed H_2_O molecule, and the number of H_2_O molecules in the cluster, respectively. As shown in Figure 3, for the pristine surface, the monomer H_2_O is preferably adsorbed at the hollow site, with two O-H bonds orienting towards the monolayer. In the case of the dimer, trimer, tetramer, and pentamer, water clusters prefer to form the linear, triangular, quadrilateral, and pentagonal shapes on the surface of MoS_2_, respectively. In particular, we observe that a ring trimer is more stable than an open structure on the MoS_2_ surface, which differs from that on the Pt(111) surface [44]. In addition, due to the cluster–surface interaction, these water clusters tend to lie on the pristine surface. Generally speaking, the shape and orientation of water clusters on the pristine MoS_2_ surface are almost identical to the gas-phase of water clusters [45,46,47,48]. As displayed in Table 1, the binding energy of water clusters on the pristine surface dramatically increases with the increasing size of the cluster as more hydrogen bonds form within the cluster. We also note that the hydrogen bonds in the cluster become stronger with the size of the water cluster, e.g., trimer: 0.23 eV, tetramer: 0.31 eV, and pentamer: 0.33 eV. This is because the angular strain becomes smaller in a large water cluster. In comparison with the adsorption energy, the binding energy has a larger negative value since it contains contributions from the intermolecular hydrogen bonding in addition to the cluster–surface interaction. Both binding and adsorption energies show that the water cluster binds stronger to the MoS_2_ surface than monomer water, indicating the tendency towards larger water clusters on the MoS_2_ surface. In general, the water clusters are weakly bound to the pristine MoS_2_ with a large separation bigger than 2.98 Å between them.

For the defective surface, we find that the monomer water adopts a vertical configuration with one O-H bond pointing towards the defect, which is in good agreement with the previous calculations [12]. When the dimer, trimer, tetramer, and pentamer adsorb on the defective MoS_2_ surface, the structure of the water clusters changes as influenced by hydrogen bonding and cluster–defect interactions. Therefore, one molecule of the water cluster is always adsorbed at the defect with a small separation. The adsorption energy of water clusters on the defective surface (in range of −0.21 eV to −0.54 eV) is slightly larger than that on the pristine surface (in range of −0.14 to −0.49 eV), implying that the defect is a center of adsorption. Although the adsorption energy and distance between the water cluster and MoS_2_ monolayer are greatly influenced by the presence of the defect, all these water clusters are only physisorbed on the defective MoS_2_.

### 3.2. Electronic Properties

**Figure 4:**
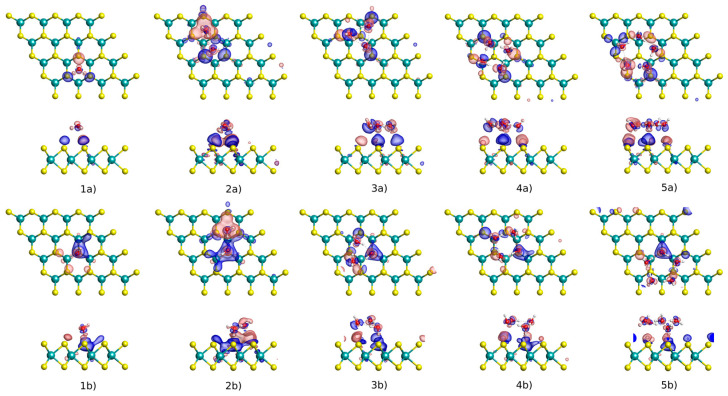
Charge density difference plots for (H_2_O)*_n_* (*n* = 1–5) on the pristine and defective MoS_2_ monolayers by vdW-DF-cx functional. The notations of (**1a**–**5a**) and (**1b**–**5b**) correspond to the pristine and defective surfaces, respectively; numbers 1–5 correspond to the number of water molecules on the surface. The red (blue) distribution corresponds to charge accumulation (depletion). The isosurface value of 0.005 e^−^/Bohr^3^ is considered for all the cases.

Figure 4 and Table 1 display how the electron density rearranges upon the adsorption of the water clusters. The corresponding charge density difference is calculated by the formula $\Delta \rho =\rho_{tot}-\rho_{{MoS}_{2}}-\rho_{cluster}$, where $\rho_{tot}$, $\rho_{{MoS}_{2}}$ and $\rho_{cluster}$ are the charge density of the MoS_2_ with adsorption, MoS_2_ monolayer, and isolated cluster, respectively. It is shown that for both pristine and defective MoS_2_, the oxygen atom in the water gains charge density, while the hydrogens lose charge density, resulting in a small number of electrons to be transferred from MoS_2_ to the water cluster. It also implies that the charge rearrangement between MoS_2_ and the water cluster depends on the orientation of the water molecule. For example, when a water dimer is adsorbed on the pristine surface, 0.022 e is transferred from the MoS_2_ to one water molecule (labeled as H_2_O(2) in Table 1); while 0.005 e is transferred to the MoS_2_ from the other water molecule (labeled as H_2_O(1) in Table 1). The charge rearrangement mainly occurs on the S atoms and partly on the Mo atoms of MoS_2_ around each absorption site. As the water cluster grows, the charge transfer between the water cluster and host tends to converge for both pristine and defective surfaces. Compared with the pristine surface, a larger charge transfer occurs between the water cluster and the defective MoS_2_, also reflected by their stronger adsorption as described above. As revealed by Table 1, up to 0.033 e is transferred from the defective MoS_2_ to the water cluster. The transferred electrons are mainly distributed in the water molecule adsorbed at the S defect (labeled as H_2_O(1)). This behavior arises from the presence of the unsaturated Mo atom on the surface, which leaves excess electrons at the S defect. We can conclude overall from this analysis that the water clusters acting as electron acceptors induce charge redistribution for both pristine and defective MoS_2_ hosts.

We next examine the band structure and DOS (see Supporting Information) of the water cluster adsorbing on both pristine and defective MoS_2_ surfaces. Since the DFT method always underestimates the band gap, the G_0_W_0_ calculation is performed for the pristine and defective MoS_2_. For the pristine MoS_2_ monolayer, our G_0_W_0_ band gap of 2.55 eV is slightly larger than our previous result of 2.49 eV, mainly due to the exclusion of spin–orbit interaction in the present work. We find that the spin–orbit splitting calculated by G_0_W_0_ is almost the same as vdW-DF-CX. Therefore, after including the spin–orbit splitting from the DFT method at the vdW-DF-CX level, the calculated band gap of 2.48 eV of the pristine MoS_2_ monolayer agrees well with the experimental values of approximately 2.5 eV [49]. The presence of an S defect on the MoS_2_ induces three localized states in the band gap: two unoccupied states around 1.58 eV and one occupied state around −0.42 eV (see Figure S1 in Supporting Information). In addition, the unoccupied states undergo a spin–orbit splitting of 0.05 eV at the K point, giving rise to a QP band gap of 1.95 eV. Concerning the adsorption of the water clusters, our DFT band structure and DOS calculation show that the molecular states are present deeply in the valence band and conduction band of MoS_2_ and hybrids with host states, implying that the band gap of MoS_2_ is insensitive towards perturbations by the water clusters. Thus, the G_0_W_0_ calculation will not be performed upon the adsorption of the water clusters.

### 3.3. Optical and Excitonic Properties

**Figure 5:**
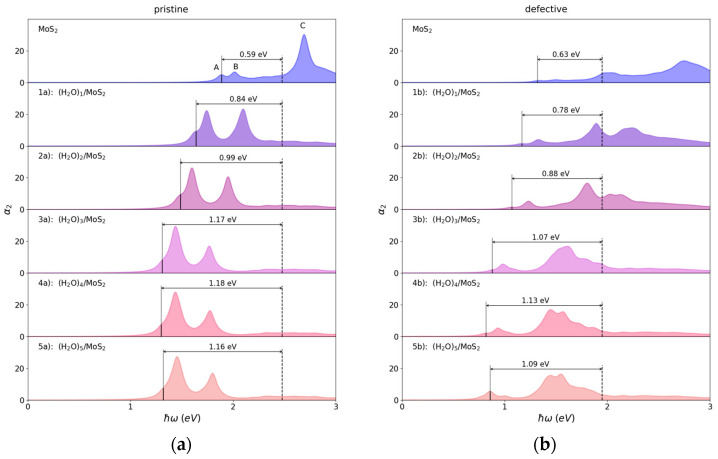
The optical absorption spectra for (H_2_O)*_n_* (*n* = 1–5) on the pristine and defective MoS_2_ monolayers simulated with the DFT-*app*G_0_W_0_-BSE method. The dark solid and dashed lines indicate the optical and electronic band gap, respectively. Excitonic binding energies are shown as well. The notations of A, B and C correspond to the optical absorption peaks. The notations of (**a**) and (**b**) correspond to the pristine and defective surfaces, respectively; numbers 1–5 correspond to the number of water molecules on the surface.

We proceed to investigate the optical properties of (H_2_O)*_n_*/MoS_2_ by solving the BSE equations. Figure 5 depicts the optical absorption spectra with and without the adsorption of water clusters on the MoS_2_ monolayer, and the corresponding results are summarized in Table 2. The calculated optical band gap of 1.89 eV for the pristine MoS_2_ monolayer is in excellent agreement with the experimental value of approximately 1.9 eV [6,49]. As a result, the corresponding excitonic binding energy of 0.59 eV is obtained in this work. The sharp peaks A and B are located at 1.89 eV and 2.02 eV, respectively, associating with the direct transitions from the spin–orbit split valence band to the conduction bands at the K point in the Brillouin zone. The adsorption of the monomer water on the pristine MoS_2_ dramatically redshifts the absorption edge of the optical spectrum and induces an absorption shoulder at 1.64 eV, corresponding to a strongly bound exciton with 0.84 eV binding energy. It is well known that the excitons in 2D-TMDs are of the Wannier–Mott type and thus delocalized in space [50]. A localized exciton with strong electron–hole interaction is observed upon the adsorption of the water cluster. In addition, as the water cluster grows, the optical absorption peak is shifted to lower energy and the binding energy of excitons becomes larger, and both of them finally converge to a value of approximately 1.32 eV and 1.17 eV, respectively. This convergence indicates that the exciton binding energy may be insensitive to further growth of the water cluster. More interestingly, the optical absorption is greatly strengthened in the spectral range of 1.5–2.5 eV. The enhanced optical absorption is attributed to the charge transfer between the water cluster and host which depletes the n-type conductivity in MoS_2_ and further reduces the electrostatic screening [51,52]. 

In comparison with the pristine MoS_2_, the presence of an S defect greatly redshifts the first absorption peak to 1.32 eV with attenuated intensity. The excitonic binding energy with 0.63 eV is slightly stronger than that of the pristine MoS_2_. This is because the unsaturated Mo atom leaves excess electrons at the S defect, leading to the formation of the charged exciton. The adsorption of the water clusters on the defective surface also redshifts the optical absorption edge compared with that on the pristine MoS_2_, but the optical band gap converges to a smaller value of approximately 0.86 eV with the increasing size of the water cluster. Thus, the binding energy of the charged exciton converges to a value of approximately 1.09 eV with the increasing size of the water cluster. Although the first optical absorption peak is weakened by the adsorption of the water clusters, the optical absorption between 1.5 and 2.0 eV is greatly strengthened as similarly observed for the pristine MoS_2_ series. In conclusion, the optical absorption of both pristine and defective MoS_2_ can be redshifted and is further enhanced in the infrared region by water clusters adsorption.

## 4. Conclusions

In summary, by employing the DFT-*app*G_0_W_0_-BSE method, we explored how the electronic and optical properties of the MoS_2_ monolayer are affected by the adsorption of water clusters of different sizes. It was found that (1) the water clusters (H_2_O)*_n_* (*n* = 1–5) have weak interaction with both pristine and defective MoS_2_ monolayers, (2) no additional states are introduced in the gap region of both pristine and defective MoS_2_ by the adsorption of the water clusters; and (3) the presence of the water clusters can dramatically redshift the optical absorption for both pristine and defective MoS_2_ monolayers. 

## Figures and Tables

**Table 1:** The binding energy (*E*_bind_), adsorption energy (*E*_ad_), and equilibrium height (*h*) between the center of mass of the molecule and the top S-layer, total charge transfer (ΔQ) from MoS_2_ to water cluster, and individual charge.

No. *^a^*	*E*_bind_(eV)	*E_ad_*(eV)	h(Å)	ΔQ(*e*) *^b^*	Individual ΔQ(*e*)				
					H_2_O(1) *^c^*	H_2_O(2) *^c^*	H_2_O(3) *^c^*	H_2_O(4) *^c^*	H_2_O(5) *^c^*
1a)	−0.140	−0.140	2.976	0.006	0.006				
2a)	−0.488	−0.273	2.866	0.017	−0.005	0.022			
3a)	−0.990	−0.308	3.110	0.025	0.010	0.000	0.015		
4a)	−1.649	−0.402	3.193	0.022	0.008	0.001	0.012	0.001	
5a)	−2.127	−0.486	3.177	0.020	0.011	0.002	0.007	−0.003	0.003
1b)	−0.209	−0.209	2.082	0.032	0.032				
2b)	−0.537	−0.322	2.385	0.025	0.018	0.007			
3b)	−1.029	−0.347	2.849	0.032	0.023	0.004	0.005		
4b)	−1.663	−0.416	2.976	0.033	0.026	0.001	0.006	0.001	
5b)	−2.178	−0.537	3.061	0.031	0.022	0.004	−0.003	0.007	0.001

*^a^* The numbering notations correspond to the structures presented in Figure 3. *^b^* The sign of the charge transfer value indicates the direction. *^c^* The numbering notations correspond to the water molecules presented in Figure 3.

**Table 2:** The QP electronic band gap (*E*_ele_), optical band gap (*E*_opt_), and excitonic binding energy (*E*_ext_) for (H_2_O)*_n_* (*n* = 1–5) on the pristine and defective MoS_2_ monolayers.

No.	*E*_ele_(eV)	*E*_opt_(eV)	*E*_ext_(eV)
pristine MoS_2_	2.48	1.89	0.59
1a)	2.48	1.64	0.84
2a)	2.48	1.49	0.99
3a)	2.48	1.31	1.17
4a)	2.48	1.3	1.18
5a)	2.48	1.32	1.16
defective MoS_2_	1.95	1.32	0.63
1b)	1.95	1.17	0.78
2b)	1.95	1.07	0.88
3b)	1.95	0.88	1.07
4b)	1.95	0.82	1.13
5b)	1.95	0.86	1.09

## Data Availability

Not applicable.

## Supplementary Materials

The following supporting information can be downloaded at: https://www.mdpi.com/article/10.3390/nano13020229/s1, Figure S1: electronic band structure for the pristine and defective MoS_2_ monolayers; Figures S2 and S3: electronic band structure for (H_2_O)*_n_* (*n* = 1–5) on the pristine and defective MoS_2_ monolayers, respectively; Figure S4: total density of states and the projected density of states for (H_2_O)*_n_* (*n* = 1–5) on pristine and defective MoS_2_; optimized geometries considered in this work.
