# Supplementary material for: Cluster Formation Effect of Water on Pristine and Defective MoS2 Monolayers
Source: Nanomaterials (Basel). 2023 Jan 4;13(2):229. doi: 10.3390/nano13020229 (PMC9864297; doi:10.3390/nano13020229)
Supplement: Supplementary file 1 [file nanomaterials-13-00229-s001.zip › nanomaterials-2100450-supplementary.pdf]

Supporting Information (SI)

Cluster Formation Effect of Water on Pristine and Defective MoS_2 Monolayers

Kangli Wang* and Beate Paulus*

Institut für Chemie und Biochemie, Freie Universität Berlin, 14195 Berlin, Germany

E-mail: klwang0329@zedat.fu-berlin.de; b.paulus@fu-berlin.de

Figure S1. Electronic band structures by vdW-DF-cx and G_0W_0 for pristine and defective MoS_2 monolayer.

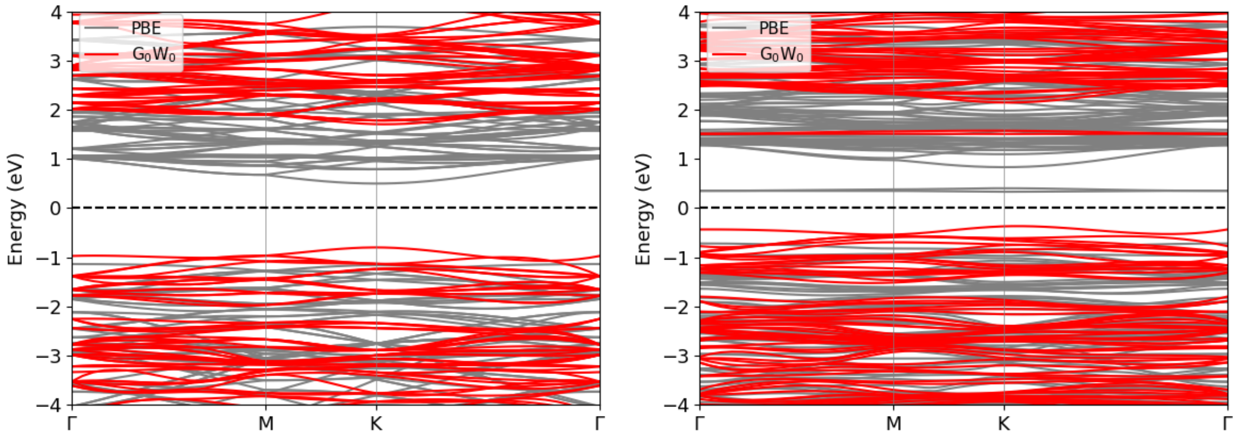

(a) pristine (b) defective

Figure S2. Electronic band structures for $(H_2O)n$ (n=1-5) on pristine MoS_2 monolayer by vdW-DF-cx functional.

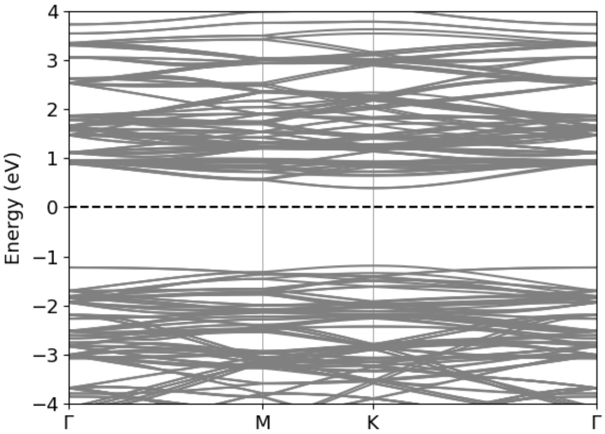

(a) $(H_2O)_1$

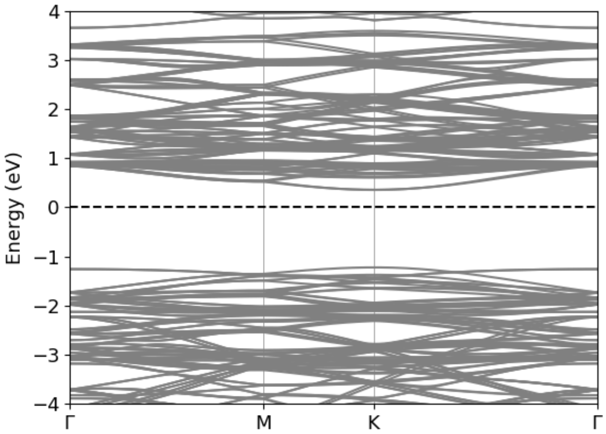

(b) $(H_2O)_2$

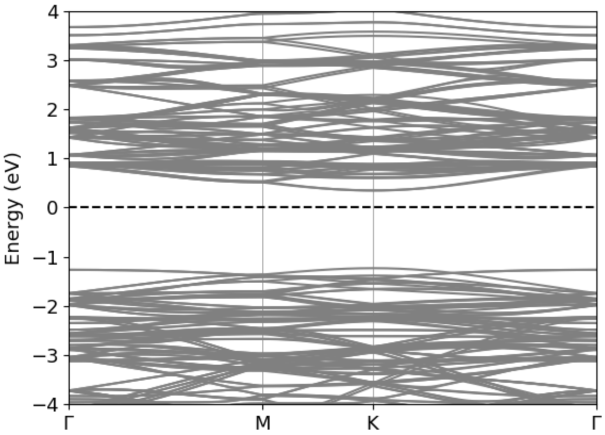

(c) $(H_2O)_3$

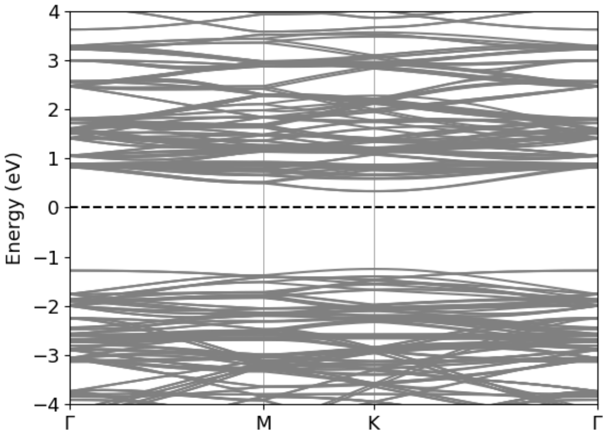

(d) $(H_2O)_4$

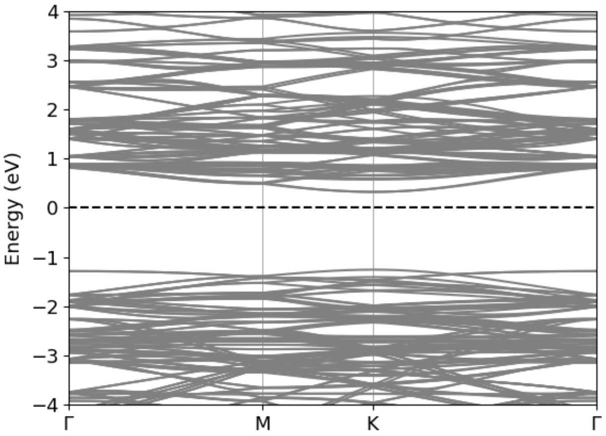

(e) $(H_2O)_5$

Figure S3. Electronic band structures for $(H_2O)n$ (n=1-5) on defective MoS_2 monolayer by vdW-DF-cx functional.

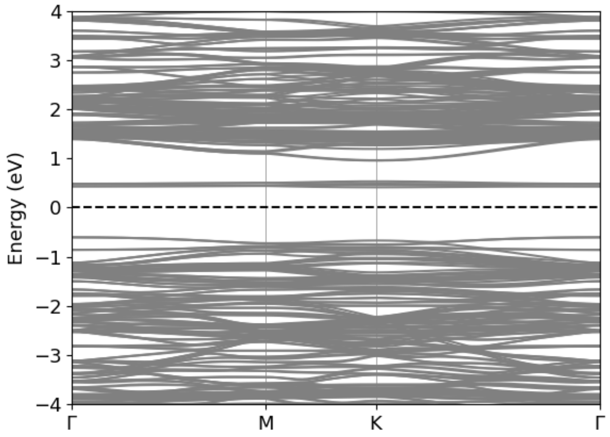

(a) $(H_2O)_1$

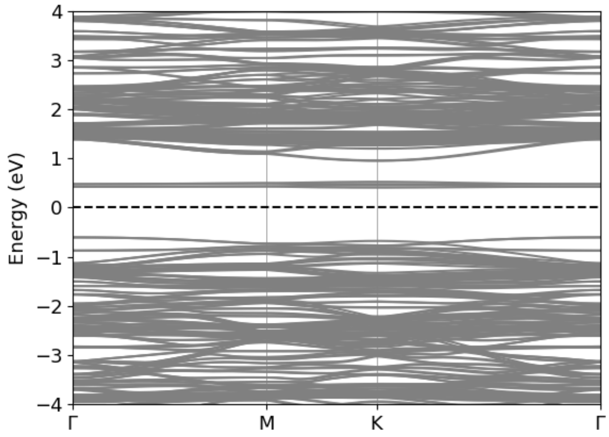

(b) $(H_2O)_2$

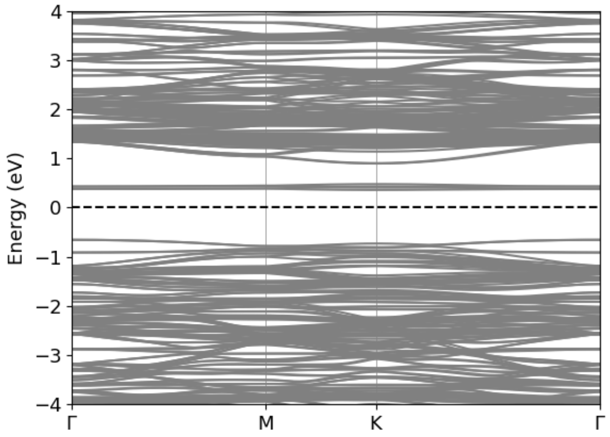

(c) $(H_2O)_3$

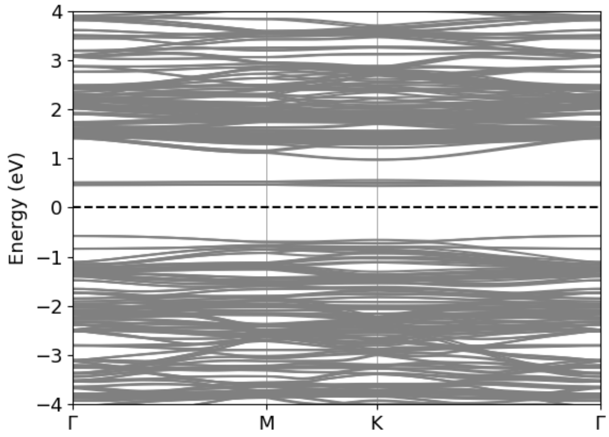

(d) $(H_2O)_4$

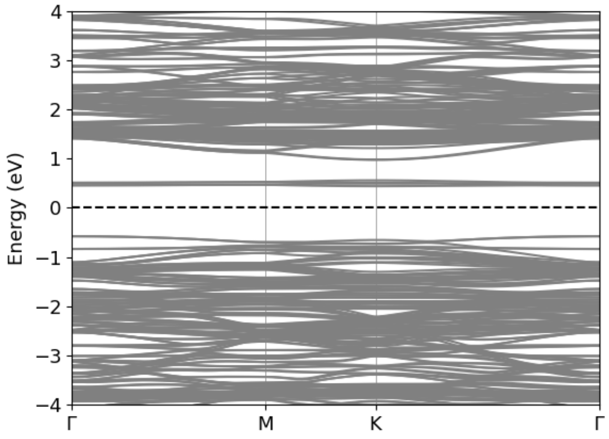

(e) $(H_2O)_5$

Figure S4. Total density of states and the projected density of states for $(H_2O)n$ (n=1-5) on pristine and defective MoS_2 monolayer by vdW-DF-cx functional.

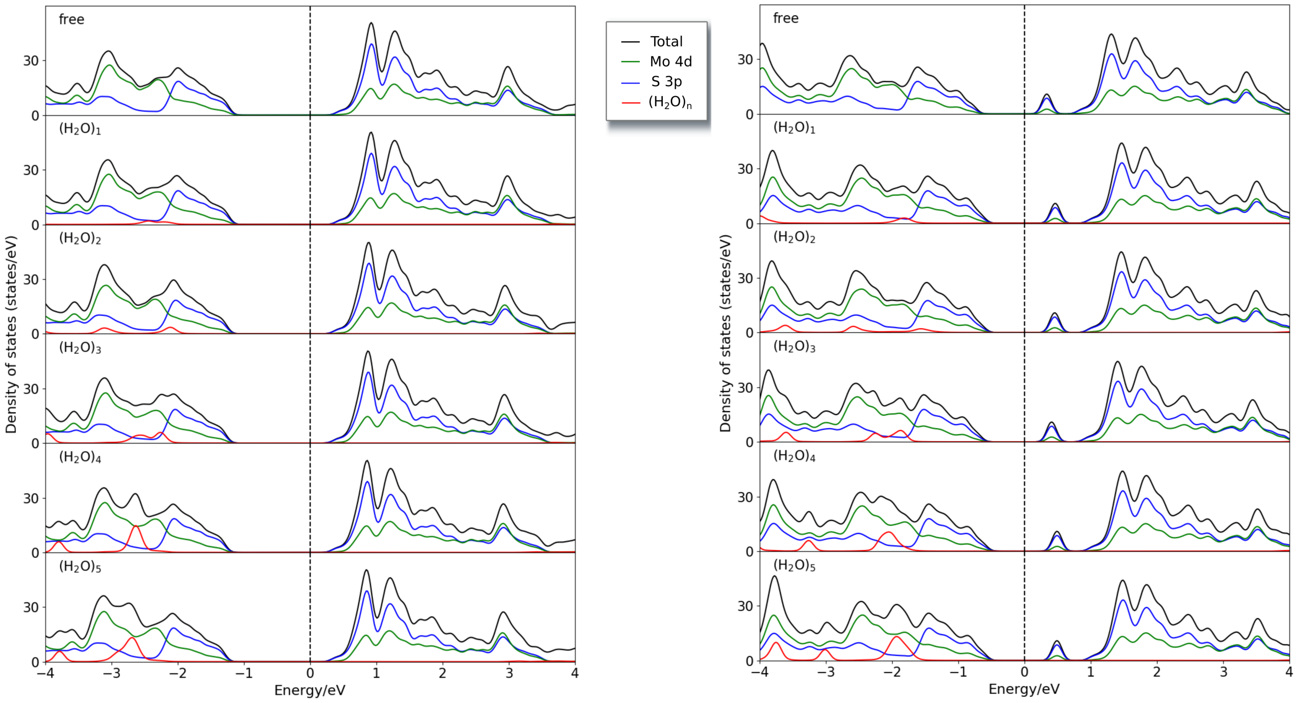

(a) pristine surface

(b) defective surface

Optimized geometries considered in this work (POSCAR format)

(a) Optimized geometry for $(H_2O)_1$ on pristine MoS_2 monolayer:

Mo S O H

1.00000000000000

12.7335259874836861 0.0000000000000000 0.0000000000000000

-6.3667629937418431 11.0275569849102020 0.0000000000000000

0.0000000000000000 0.0000000000000000 21.1729065632692084

Mo S O H

16 32 1 2

Direct

0.5005130430624831 0.2498087961612470 0.4274562637848121

0.5003902429742766 0.7499699793269627 0.4274704200367694

0.0002040590848225 0.2498566841930909 0.4275955905225572

0.5004129689188801 0.9999216811120348 0.4276561802671068

0.7503456862193545 0.9999478607999208 0.4276709583042617

0.7503788561748124 0.2498964890202657 0.4276726767268073

0.7504082284357452 0.4999904263967778 0.4276813359063851

0.7504221723760551 0.7499985481206153 0.4277012754927938

0.2503867283618249 0.7500059902041230 0.4277022296367079

0.2503830255654620 0.9999004142076515 0.4277041406638915

0.0003939201375474 0.4999839096315384 0.4277062345190714

0.2503943461173179 0.2498751549205807 0.4277160828598952

0.5003675104895748 0.5001455799817762 0.4277203544982555

0.0003404101698905 0.9999143258111189 0.4277293223269965

0.2506440309247964 0.5001940536374292 0.4277305820973538

0.0003914046148878 0.7499809115760669 0.4277314121676099

0.4170512265374455 0.5832721338734856 0.3538098260441842

0.4169478771790054 0.0830008926049928 0.3538484379842828

0.6673222494262419 0.3333791975893305 0.3538564487408351

0.4169490901305366 0.8333841143215679 0.3538591119557282

0.6672593329501240 0.8334071198703370 0.3538639852120085

0.9169614153826586 0.0831427167644279 0.3539166742935365

0.4170533079563583 0.3333447215036003 0.3539178311965756

0.9169830892809969 0.3333365307501879 0.3539179139910686

0.6670484133887626 0.0832663503128117 0.3539193530943834

0.1669822402951908 0.8332644399456584 0.3539610877517063

0.9171049752732188 0.5833761752311091 0.3539612103139973

0.9170897983547022 0.8332942393835978 0.3539659836784708

0.6670268757312527 0.5833270103072081 0.3539863242490355

0.1670953317123889 0.5833256231459316 0.3539934063187360

0.1671166391094729 0.3333450345375795 0.3539948166116460

0.1670247863236298 0.0832550399201111 0.3539986916582620

0.4174229017854714 0.3331045848166099 0.5011596588447631

0.1668142605474827 0.3332349196679232 0.5012620906952705

0.6669840296749712 0.3332172421351487 0.5013378039656828

0.4171697596055708 0.0833230985119684 0.5013565746525899

0.6668845792779976 0.8332205700295319 0.5013658312938247

0.4171417584001489 0.8332257485110475 0.5013709165663514

0.4173534977289819 0.5838383581975606 0.5014046899171163

0.9170189269514069 0.3332589248941389 0.5014062659879244

0.9169486265894804 0.0832669201579748 0.5014209668509864

0.6670030751244411 0.0832216572769298 0.5014654397247114

0.9170379939367876 0.5833012482095441 0.5014693244062869

0.1670605754751762 0.8332795792009051 0.5014761486937118

0.1670441556001023 0.0832208214617083 0.5014810615761078

0.9170125309346560 0.8332581019144740 0.5014833989972800

0.6670331820230970 0.5833402987445453 0.5014839685931420

0.1671354219822234 0.5833743385595724 0.5014871188455530

0.3425268697175596 0.4204220439163535 0.6433309469957251

0.2579256852375167 0.3717525896814351 0.6302465592111659

0.3778248896119578 0.3702441059780455 0.6321601982233176

(b) Optimized geometry for $(H_2O)_2$ on pristine MoS_2 monolayer:

Mo S O H

1.00000000000000

12.7335259874836861 0.0000000000000000 0.0000000000000000

-6.3667629937418431 11.0275569849102020 0.0000000000000000

0.0000000000000000 0.0000000000000000 22.2171660419423347

Mo S O H

16 32 2 4

Direct

0.5012764434112498 0.5020768547898911 0.4262267256374201

0.2511943660525020 0.2517387147306209 0.4266621034431139

0.2513428092737584 0.5022138995589245 0.4267460632963420

0.5011695936116851	0.2517270490461740	0.4267576705373131
0.5011197332939759	0.0018207842432787	0.4267646380326156
0.7511058008860090	0.5017731790977535	0.4267707049179990
0.0008195177603056	0.2515848088381105	0.4267788710810336
0.7511064723298659	0.7519397501501572	0.4268116035635927
0.0011782172705850	0.5017244364795346	0.4268153932008403
0.5011169536784266	0.7521174317529500	0.4268310217917843
0.7511471835227823	0.2517544075989733	0.4268562135736218
0.0012120808673615	0.0018604842421652	0.4268874961862963
0.7511846368701285	0.0018226649862285	0.4268985893202952
0.2511102218250514	0.0017352083536011	0.4270015551079069
0.0011163633293023	0.7517735216733996	0.4270471981929944
0.2512353010524748	0.7518377097797000	0.4271403786191570
0.4177898717521700	0.5857387848313707	0.3562318519409189
0.4178076879281889	0.3349218650199575	0.3562852367874711
0.6683434449696435	0.5855469980192183	0.3563079833429512
0.9177610669792671	0.0849276904468965	0.3565289258785924
0.6678473249287720	0.3349877667424366	0.3565376082640768
0.9176871179143049	0.3350576287255578	0.3565437899705870
0.1677947500523373	0.0848397640819485	0.3565596918637155
0.1677582214356548	0.3352632900522821	0.3565616127657734
0.6678319888702191	0.8353990283256749	0.3565821647569294
0.4175435980092885	0.0849310820412512	0.3565922578083871
0.1678066386728361	0.5855204516136610	0.3565994262744141
0.4175184429835355	0.8352172746514270	0.3566069780476511
0.6679151654180373	0.0851494982329157	0.3566124708724274
0.9179232958292332	0.8351613755977354	0.3566631789149284
0.9179404163801763	0.5852837304610432	0.3566914218509893
0.1677988978874296	0.8349870986474883	0.3568404869024846
0.4184997867643787	0.5853292970358268	0.4965283407062131
0.1676809107597066	0.3351256380771375	0.4966844110165951
0.4181266899787559	0.3356671715290318	0.4967379795049283
0.6675059314471525	0.5850886465450955	0.4969107670689610
0.6677306204498592	0.3350858505702021	0.4970846425305169
0.9177426946293963	0.3350239276981668	0.4971498556183249
0.1679108704310579	0.0853320800144246	0.4971549989385764
0.9178165937496203	0.0850657985520868	0.4971666498394853

0.6677345064600004 0.0850869102997081 0.4971724912685715

0.9176421618913935 0.5848578970687157 0.4971844806464958

0.4179229744631598 0.0851820824218237 0.4971850878346444

0.9176102487317692 0.8351745682749723 0.4972056737185113

0.6675889571005555 0.8350942251852729 0.4972244816978559

0.4182319014948845 0.8356607961863105 0.4972649206988677

0.1676603526006915 0.5849096321901754 0.4972695914115874

0.1678090821925835 0.8350955134125755 0.4974393695908930

0.3594107631835612 0.6688243764319139 0.6179768132479424

0.3146081286305389 0.4249266144271147 0.6340066902956139

0.2513850193792067 0.3745893350315441 0.6048912779708004

0.3857455299679771 0.4216881905360523 0.6200498535228860

0.3426892808030502 0.5860351958138281 0.6276333697860323

0.3401695084552614 0.6974089490105371 0.6547965362700339

(c) Optimized geometry for $(H_2O)_3$ on pristine MoS_2 monolayer:

Mo S O H

1.00000000000000

12.7335259874836861 0.0000000000000000 0.0000000000000000

-6.3667629937418431 11.0275569849102020 0.0000000000000000

0.0000000000000000 0.0000000000000000 22.2171660419423347

Mo S O H

16 32 3 6

Direct

0.5026397567011074 0.7510055755170910 0.4227456700494159

0.5028958203581197 0.5013527167046448 0.4228055523043110

0.2527104786301066 0.5010691827356695 0.4228368237994573

0.7531542910717093 0.0014937216849162 0.4228592620797968

0.0027786680509934 0.5010411967546915 0.4228809200175832

0.7530542691753226 0.7509900555791518 0.4228885235075950

0.5028334029202028 0.0008897388617797 0.4229604308785397

0.7527006759253112 0.5010077651432780 0.4229727012103837

0.0029120165906917 0.2511175778814234 0.4229765158283172

0.2525509633099361 0.2509492360626027 0.4229945344630650

0.7528448445008920 0.2510731521758274 0.4229960840306362

0.2527381116997987 0.7508363732175667 0.4230139609731438

0.0028420882813478 0.0010183344339438 0.4230209455723326

0.5029410601465401	0.2508514430517366	0.4230308524614301
0.0027982116553460	0.7510225160779314	0.4230479978974984
0.2528552702106808	0.0009676668200029	0.4230834677228827
0.4193865135506343	0.5843917959180231	0.3524253815819023
0.1693361335577848	0.5843711539027225	0.3525804784557991
0.6696031552265325	0.5844620366795112	0.3525864420876914
0.4192897336446038	0.8343354569457162	0.3525970266059701
0.1694028352239059	0.3342726201319195	0.3526184480149439
0.6697795575909282	0.8345984919954574	0.3526244158777985
0.9198008577989114	0.0845475128603539	0.3526357106126667
0.6695303151252858	0.0845148639245608	0.3526447126608900
0.9197280697478050	0.8344573552956263	0.3526680261203552
0.9194481814038582	0.3343102883117908	0.3526683174716467
0.9193761642676748	0.5844306815932967	0.3527065144225290
0.6694525225269032	0.3342977170777246	0.3527180033765873
0.4194566233944457	0.0842375647000608	0.3527592269961204
0.1694994043415647	0.0842474616337299	0.3527709224571680
0.4194028513379564	0.3341926298855569	0.3527894102336546
0.1694584460310935	0.8343167674934406	0.3527935704402196
0.6696861714840381	0.8346439032321129	0.4927696110895567
0.4193550734889513	0.5845504308724756	0.4928712356620011
0.4194894903321398	0.3344392760832378	0.4930155051873726
0.1692146757908191	0.5841136127262061	0.4932362529636976
0.6695772723789162	0.0843977591114253	0.4932421137471934
0.6694188552429878	0.5844221178651665	0.4932665453060565
0.9196017005219659	0.3345168122048960	0.4932681175137787
0.9196162062098026	0.0845401565619355	0.4932684159316807
0.9194224228663614	0.5842492833014816	0.4932900739594928
0.4194478747105350	0.8341565725379851	0.4932970248910209
0.6695468808434839	0.3343796629702922	0.4933179479015948
0.9195015645715472	0.8343382428755532	0.4933199931669492
0.1695321829249039	0.3346170605193279	0.4933241314085137
0.1694578927059851	0.0843333694192268	0.4933395226604560
0.4195817161822717	0.0841703381147880	0.4933401307488197
0.1694849827747191	0.8341817706848076	0.4933663474138470
0.3172706097162461	0.6130833996370484	0.6328924055145038
0.5678122251952047	0.7068147898637633	0.6339931678688799

0.4104412642480852 0.4592678525198508 0.6345112875056671

0.6132187330173124 0.7615612257993121 0.6009625905839755

0.4119452403385822 0.4088378390761704 0.6017528988513234

0.4870414774582770 0.5381926951836391 0.6307869776704820

0.3254271584270043 0.5392207990524795 0.6334346483900006

0.4876178212546378 0.7022248628228738 0.6340093116920542

0.2755867985112133 0.6099935671341044 0.6702560935039088

(d) Optimized geometry for $(H_2O)_4$ on pristine MoS_2 monolayer:

Mo S O H

1.00000000000000

12.7335259874836861 0.0000000000000000 0.0000000000000000

-6.3667629937418431 11.0275569849102020 0.0000000000000000

0.0000000000000000 0.0000000000000000 22.2171660419423347

Mo S O H

16 32 4 8

Direct

0.7487367619058602 0.4971323750583778 0.4192888682298204

0.4989632192264963 0.9975875872565041 0.4194337373366182

0.2483925919306031 0.7469750012873817 0.4194595899137994

0.7487327989181480 0.9970777947423812 0.4194852404850309

0.4989739743009949 0.7471846075495137 0.4195180947202886

0.9988007087627011 0.4972069659220750 0.4195299456917212

0.4988682199493013 0.2471410292170191 0.4195629734864710

0.7489344729893759 0.2471798724531524 0.4195717152404654

0.2487344694211799 0.9972028810150633 0.4195976434348623

0.9985681314017256 0.7471826235198620 0.4196073880620332

0.4989565868800341 0.4974545821442149 0.4196446612677391

0.9985994747162721 0.9970562582406615 0.4196562498294654

0.9988133953255556 0.2472012143514846 0.4196570146884895

0.2487702855174589 0.4973249647682181 0.4196637364278715

0.2485421685678162 0.2471704874189058 0.4196856662275223

0.7486276323051229 0.7469821772194720 0.4197756801832284

0.9156402252472873 0.5806910894917650 0.3491792871041497

0.6654470174558398 0.3302313279344773 0.3491945547579718

0.6657123173492749 0.0806472726658285 0.3492028638285163

0.1653208089731280 0.5803252996794299 0.3492140851966994

0.4153030176842591	0.0806569671067763	0.3492141802583291
0.6653340460649204	0.5806812382617821	0.3492271580161272
0.4154731378276679	0.8305892452456547	0.3492636038435819
0.4154402190098310	0.5805242870891547	0.3492942609489305
0.1651433084031666	0.8305449215572196	0.3492955299676694
0.6656313919666772	0.8303369869255377	0.3492988880467323
0.9155761343704754	0.3304621837807389	0.3493077034374750
0.9155024339055302	0.0805425416722301	0.3493186575348446
0.9150716528225544	0.8303851752958806	0.3493940778650853
0.1653006521931957	0.0805520731577758	0.3493996887067112
0.1654320933802182	0.3305802658728823	0.3494083455263848
0.4153616102004634	0.3306391819139307	0.3494288765427669
0.4154318899246690	0.8308062600389832	0.4894735799998244
0.6656849018168955	0.3307167830545268	0.4897548971573826
0.9151488345937793	0.5803681789862978	0.4897840246347016
0.6655103714056025	0.0804701382377147	0.4898252657182098
0.6657019993782782	0.5801249656432503	0.4898273087330551
0.4154840337490882	0.3305312109461482	0.4898345000192705
0.1652448724358209	0.8303618634236045	0.4898390559558763
0.4155186605428867	0.0805915666739310	0.4898431644906864
0.4155569925220703	0.5807410384515137	0.4898712162232428
0.1652461429683640	0.5805774789249156	0.4898860916578229
0.6654634693230506	0.8306233856269287	0.4898888543864217
0.9152492505749876	0.0803824420234633	0.4898914726321451
0.9155571033608894	0.3306241634469984	0.4899373736259136
0.1652591570561839	0.0804001701567501	0.4899378880929675
0.1654314664315777	0.3307065369047990	0.4900048912380655
0.9152690132965091	0.8304268040991261	0.4900663122130666
0.6011762081985452	0.6557580909257865	0.6252616239971829
0.2592096048592154	0.5293256043915662	0.6333179719428301
0.4575571713732813	0.7520653528563486	0.6340869449057962
0.4042994395132240	0.4338213574907215	0.6415149003744816
0.4530275337966359	0.7988793215083732	0.6003195229680145
0.4007992954875448	0.3799148540998587	0.6093028406253254
0.5564879218008514	0.7024777443998005	0.6279174897313027
0.3771835903258349	0.6744772760800686	0.6345032509271178
0.4839270984834201	0.5109256609337649	0.6360260351533427

0.3041682956784300 0.4830622584212705 0.6367259007424337

0.6614026817857805 0.6877857719872651 0.6573893485077065

0.2051457706047870 0.5048115130683541 0.6680145317355937

(e) Optimized geometry for $(H_2O)_5$ on pristine MoS_2 monolayer:

Mo S O H

1.00000000000000

12.7335259874836861 0.0000000000000000 0.0000000000000000

-6.3667629937418431 11.0275569849102020 0.0000000000000000

0.0000000000000000 0.0000000000000000 22.2171660419423347

Mo S O H

16 32 5 10

Direct

0.2489531089600021 0.7458827024084016 0.4188472050660863

0.7488381218714224 0.4957759513431768 0.4189574962670122

0.9988466474532416 0.4958293102438347 0.4190218391699148

0.2486356284028517 0.2457422948885792 0.4190220420878887

0.4990167953337306 0.9959918840292659 0.4190221682230586

0.4990996478986034 0.2458015044906077 0.4190613618410239

0.7489628126191192 0.2457703909542417 0.4190759726398241

0.9989525499892267 0.2457907410087543 0.4190915613574262

0.4990653312379294 0.4961868667587765 0.4191229593285968

0.4990930088695862 0.7459674571979491 0.4191298411211477

0.2489515610126176 0.9959500772819752 0.4191360744677652

0.7488742055719300 0.9957171522215589 0.4191462632577156

0.9988723012618621 0.7458320212327862 0.4191553358523095

0.7488395514034210 0.7457623722844673 0.4191776440393724

0.2489745586400716 0.4957790276278615 0.4192082133536559

0.9988378614929658 0.9957732383204885 0.4192337934521930

0.4158316504348321 0.8293147292524736 0.3487148697130849

0.1656019529928727 0.5789832982693284 0.3487480425562808

0.6656002202389644 0.3290003679322666 0.3487562010913550

0.6655248355115191 0.5792673312549326 0.3487839162213007

0.9156823567674977 0.5792985497133500 0.3487883514045578

0.1654946846978831 0.8293122760362905 0.3487986597150510

0.1655607909449088 0.3292627443223850 0.3488026183822441

0.6657025144595892 0.0791337704070827 0.3488058691356954

0.9156514376947058	0.3291458999133638	0.3488126287501316
0.4155602789629356	0.0792387153560981	0.3488220803909670
0.6656659073108315	0.8291258476909391	0.3488272231536556
0.1653544043371795	0.0790513255358647	0.3488620895383221
0.4155774191616075	0.5792947452698485	0.3488691222468887
0.9155858146971951	0.0790446110150640	0.3488717162866877
0.4156391564568693	0.3293220273368576	0.3488803407109913
0.9155516980648812	0.8291923483821861	0.3489501225726102
0.4156007390374299	0.8294123550284951	0.4890974907492165
0.4155825130025832	0.3291220779462805	0.4891030191583923
0.1654235663195323	0.5791855381578301	0.4891843316568938
0.1654600704123581	0.3289053095462293	0.4893075689478366
0.9153917826750799	0.5790569205516718	0.4893361074854212
0.6657007668271504	0.3292035779134750	0.4893462970422462
0.4157205527111074	0.0792223040164046	0.4893467321533578
0.6656781803364211	0.5789858772593348	0.4893585924303707
0.1657206419890471	0.8291377569891267	0.4893594292787782
0.9156304503678001	0.3292095506335571	0.4893721977871763
0.6655220562763446	0.0791266271643991	0.4894288563545928
0.1655827820980420	0.0792595835973060	0.4894332852264611
0.6656882576534073	0.8291728463888859	0.4894423744007668
0.4157453857267441	0.5795286277067291	0.4895095813431354
0.9154304964311066	0.8290180674584420	0.4895124745467925
0.9155102722504509	0.0791444179618281	0.4895249028607580
0.2341311872566934	0.4640374672501935	0.6257744767024676
0.6223636050206096	0.6750259793538049	0.6330516400703772
0.5349520386517312	0.8268028912800176	0.6334048757400552
0.4363849478953341	0.4499553146130424	0.6338566701952786
0.2930093556616740	0.6949528526249367	0.6339838317576465
0.4335141231249580	0.4010733853489086	0.5992613518512826
0.2654914301741371	0.7222164842642727	0.5994878921436992
0.2535412286427388	0.5512364321532743	0.6291036769299723
0.3586898446131883	0.4527477566288880	0.6317036906393483
0.5533278460185329	0.5879902187555146	0.6335331279351095
0.3841750678948799	0.7472308295508299	0.6338715578067706
0.5719236811801096	0.7728711115117903	0.6356592891815396
0.1732289560454277	0.4199051170243635	0.6566106631962754

0.6798008423100796 0.6791298968030759 0.6632542562849792

0.5685264430545658 0.8823768790517903 0.6673516853629380

(f) Optimized geometry for $(H_2O)_1$ on defective MoS_2 monolayer:

Mo S O H

1.00000000000000

12.7335259874836861 0.0000000000000000 0.0000000000000000

-6.3667629937418431 11.0275569849102020 0.0000000000000000

0.0000000000000000 0.0000000000000000 20.3797881478684886

Mo S O H

16 31 1 2

Direct

0.4987104480172562 0.4970976913172009 0.4640276768240312

0.4987594091452934 0.2538452658611163 0.4641316345995676

0.2552468459268056 0.2537664985231842 0.4641458247366188

0.7482450119688764 0.5001884468209425 0.4656594174554911

0.2523695533309862 0.5000717595847490 0.4656830772795999

0.2524403020284609 0.0045694107868357 0.4657172670145400

0.2515236075107410 0.7518760639315687 0.4668196484754219

0.0007606281990817 0.5010753583589960 0.4668247106672609

0.0007534416352897 0.7519054435885764 0.4668334373744116

0.5009355254676393 0.7506949587686478 0.4672458180566181

0.5009375835299466 0.0024945491209678 0.4672761775914225

0.0018959494771522 0.2516661837677105 0.4673155326726786

0.7501595746670304 0.7507618688361504 0.4673318428634872

0.0019108826779686 0.0024729599789239 0.4673392090812385

0.7501452070739916 0.2516465683745608 0.4673531319780224

0.7510151804677250 0.0016255029569621 0.4678617306902737

0.4174815292203533 0.3348006659549370 0.3870933179068530

0.4196135112531039 0.5852523860979204 0.3890235194295784

0.1672181404539046 0.3328424798684182 0.3890468332875514

0.4196510537220632 0.0866435252624029 0.3890751304275923

0.1672186652902568 0.0865650501200506 0.3890791858104876

0.6660596452792618 0.5853334664332763 0.3890844274899550

0.6660006312115172 0.3328726191543723 0.3891030273317995

0.9174920218210332 0.5848510843767087 0.3902020074744854

0.1678108919730548 0.5847718140833393 0.3902286108138924

0.1678252429972389 0.8352878752363040 0.3902656114499194

0.4186365462651054 0.8354700860814006 0.3907067110214868

0.9171598416773250 0.8354743478370068 0.3907623382243344

0.9171186420881696 0.3339029495124279 0.3907639472378293

0.6677957071512637 0.8351387149795571 0.3911059188484813

0.6677941338577753 0.0848926450628014 0.3911397423202274

0.9174887839698087 0.0848441587492559 0.3911519474132277

0.6617879539117624 0.5786828213509310 0.5423053614932769

0.4173379810137874 0.5787355134459276 0.5423054523331814

0.6617626682679756 0.3352983786594166 0.5423491732313650

0.1737295630820981 0.3351535050834258 0.5423648139924211

0.4173979274978024 0.0908474961552059 0.5423654763467525

0.1737734694324544 0.0908573681341096 0.5423961294124240

0.9158195105879656 0.5840078912294331 0.5427003050742201

0.1686689172968272 0.5839391469069781 0.5427095497725247

0.1686884612825423 0.8369911224975652 0.5427290912617906

0.4173095021123601 0.8347749428107463 0.5438081457015826

0.9179042259315509 0.3353319164021542 0.5438763966544542

0.9179031413969412 0.8348106937201365 0.5438789642761179

0.6671564988436884 0.8340544997914492 0.5441734567577328

0.6671831067789640 0.0853789351395662 0.5442186938357736

0.9186182392828215 0.0854398779656833 0.5442459531058219

0.3955634876241021 0.3180466996198064 0.6464217681641600

0.4206539780493372 0.3395659170529726 0.6006061055398249

0.4629519670047841 0.3809005089702282 0.6715778153331371

(g) Optimized geometry for $(H_2O)_2$ on defective MoS_2 monolayer:

Mo S O H

1.00000000000000

12.7335259999999995 0.0000000000000000 0.0000000000000000

-6.3667629999999997 11.0275569957496469 0.0000000000000000

0.0000000000000000 0.0000000000000000 22.2171659999999989

Mo S O H

16 31 2 4

Direct

0.5106196391927398 0.5044137857855020 0.4212884361109133

0.2669804114322787 0.2604392795252579 0.4215322582684422

0.5104457759197700	0.2603491042497694	0.4215443586476582
0.7597711140203458	0.5065792575497454	0.4222514831076438
0.2638389510845869	0.5067214306594252	0.4223282286044565
0.2640495647837966	0.0110381995711251	0.4230473482899129
0.0123530224951409	0.5077147048299082	0.4237006837375077
0.2631278082044801	0.7584935235445585	0.4239003888088249
0.0123214509925447	0.7584763036413307	0.4239051885587131
0.7617054277068291	0.2581120255838769	0.4242090021260410
0.0133037242024869	0.2581095821650052	0.4242541259328547
0.5125217505491406	0.7573205924844757	0.4242802081618464
0.7616212529609570	0.7572597752117645	0.4242947648084510
0.5125062408423702	0.0090184923518137	0.4244158431568934
0.0134150070187928	0.0090546106531164	0.4244748424197695
0.7624480116166623	0.0079829012718804	0.4247736715466388
0.4292975082337946	0.3416141815186720	0.3510093475474108
0.4313803580126461	0.5921253397708881	0.3522251190461390
0.6776144693992023	0.5921050879890188	0.3522392470305107
0.6772286779009065	0.3390186946004334	0.3523788843724134
0.1787723694006189	0.3391047687258961	0.3524502305814892
0.4311565715451380	0.0932094838739701	0.3527111954044813
0.1789872085252995	0.0932037690240790	0.3527725093643781
0.9293409078222723	0.5916972187771208	0.3533783288406482
0.1793450849267231	0.5917001792170780	0.3533894713011918
0.1794949585226533	0.8420459923538743	0.3537165052478670
0.9287352138744893	0.3404921622869708	0.3539767054253815
0.4301591105938556	0.8421344860199298	0.3540997061036961
0.9288649787062866	0.8421538733476766	0.3541578033318800
0.6792760097317441	0.8416173456521534	0.3543649221035992
0.6791352480078956	0.0912187964930027	0.3544255270069883
0.9290503880992560	0.0912485318206890	0.3544608561431559
0.4283459983210776	0.5854087335656999	0.4926259911554212
0.6737789968024330	0.5851222742828810	0.4926424308334205
0.6738711232013443	0.3421753405863726	0.4930263196042830
0.1852665945241441	0.3423050247998560	0.4930410743926004
0.9269349392657489	0.5902741293530127	0.4932808380976823
0.1803047967908142	0.5903703975210846	0.4933106145331649
0.1853303771959958	0.0973077201485495	0.4933373045029157

0.4290564668280510 0.0973216486709774 0.4933390241781481

0.1801620540820608 0.8432976731255621 0.4935824955121788

0.9293779950949030 0.3419256663313419 0.4943968704798962

0.4289238558879589 0.8412327146416843 0.4946175678755225

0.9291884628403650 0.8411686697775878 0.4946176025693489

0.6788997175384424 0.0919071319856855 0.4948720179868644

0.9298373223206085 0.0919070049656909 0.4949032721740281

0.6786507425907971 0.8405180000809764 0.4949171809190034

0.4431218801627281 0.3670799146155090 0.5911380716425683

0.5665497390545013 0.6280640493920089 0.6116796537954485

0.4344665966692105 0.3521992913454426 0.5475099170608502

0.3604527530601516 0.3197631111722714 0.6065442016679725

0.5246083285989300 0.5387569497339513 0.6080793519221004

0.6421544429371124 0.6492257839623718 0.6323962614808494

(h) Optimized geometry for $(H_2O)_3$ on defective MoS_2 monolayer:

Mo S O H

1.00000000000000

12.7335259999999995 0.0000000000000000 0.0000000000000000

-6.3667629999999997 11.0275569957496469 0.0000000000000000

0.0000000000000000 0.0000000000000000 22.2171659999999989

Mo S O H

16 31 3 6

Direct

0.5080797555457579 0.5018627986783386 0.4038328545432819

0.2643181314887784 0.2584380473046011 0.4039028485556884

0.5080318918254036 0.2585477181146274 0.4039504744199647

0.2615849019348317 0.5049719534019417 0.4049420730014859

0.2616295918953426 0.0092151373420748 0.4052154804828803

0.7572643510240837 0.5049421087873966 0.4053141142245735

0.2606583280230979 0.7566415856122006 0.4063695136733472

0.0099177677068383 0.7566313261116591 0.4064075063868060

0.0098470349834372 0.5058080537051524 0.4064107613146319

0.5102811508551923 0.7557506483196974 0.4067168943180164

0.7591991693899516 0.7554032177607226 0.4068391078191951

0.0110100849627770 0.2563795349314333 0.4068597956172937

0.0109641707652059 0.0072860593832402 0.4069089961265249

0.5101378201936484	0.0072335701791530	0.4069337179175321
0.7592834274549674	0.2564118361980263	0.4069435061636284
0.7602873800301850	0.0064676351422435	0.4074667580378915
0.4267031082582093	0.3394701305523284	0.3333628478145201
0.1762011282526856	0.3373980448448606	0.3348805393164014
0.4291652793130112	0.5902468353899479	0.3349080159421547
0.6751917534973728	0.5900641520318430	0.3349838488682835
0.1762730990781876	0.0912351171922126	0.3350285612503612
0.4289808384545495	0.0913327846483796	0.3350743764271158
0.6752087128868283	0.3375984212785141	0.3350845094683237
0.1767770885932052	0.5896876871639520	0.3360079170102779
0.1769320995898980	0.8399345549572885	0.3360880186965360
0.9265388569094881	0.5895720460534974	0.3361247798219580
0.4277592676456123	0.8403773256993006	0.3365843235394621
0.9262254931216560	0.3386912422087747	0.3366423019464122
0.9263584014103614	0.8402272387262926	0.3366497142724754
0.6771818359382493	0.8402122571896911	0.3369708569361052
0.9265174563031806	0.0895841882732427	0.3370150463540824
0.6769429726063123	0.0895850092942965	0.3371221470932895
0.4265183476985328	0.5836700137503641	0.4752904629923691
0.1831544167304813	0.3403967510428458	0.4752946438506385
0.1829949065631311	0.0953884465303929	0.4755501749452762
0.6708473678716302	0.5833483653583045	0.4756943750382945
0.4263644572906742	0.0955447343836724	0.4757093158485688
0.6709653360732588	0.3401566667394604	0.4757372891797331
0.1779317703846957	0.5885629040209110	0.4758974017535067
0.9248145667966696	0.5887042896673478	0.4759838414110433
0.1778516380972803	0.8417452819977171	0.4759905809436376
0.9268847753129776	0.8394856338551051	0.4770294745429311
0.4264643289246379	0.8394316003564057	0.4770312512267836
0.9270699526896209	0.3400556976315841	0.4771081262482113
0.6763816397502680	0.8387893970841844	0.4774257516732661
0.6764276553832360	0.0901791487296251	0.4775365067758628
0.9278554930533218	0.0903171738285948	0.4775815327293955
0.4170608660428101	0.3294854372383611	0.5809420760437689
0.1678355701277781	0.2262960120329396	0.6086330173739825
0.3248490648977551	0.4692018523723718	0.6258832628107598

0.4285442044020087 0.3282996421389939 0.5374001283733776

0.3304194350714411 0.2685343003751299 0.5876870393706000

0.3314158300289520 0.5341573106689950 0.6000369818570377

0.3815172962429259 0.4450563880622340 0.6082199494586504

0.2025593679463213 0.3138654917625345 0.6185487961099199

0.1335220450385748 0.1830816826041115 0.6462376048968750

(i) Optimized geometry for $(H_2O)_4$ on defective MoS_2 monolayer:

Mo S O H

1.00000000000000

12.7335259999999995 0.0000000000000000 0.0000000000000000

-6.3667629999999997 11.0275569957496469 0.0000000000000000

0.0000000000000000 0.0000000000000000 22.2171659999999989

Mo S O H

16 31 4 8

Direct

0.5008802167746040 0.4999608279114156 0.4039946464864954

0.2571580897963202 0.2563197293282258 0.4041470946056265

0.5006721728746655 0.2563963662866513 0.4041784019732191

0.2543145903035580 0.5026688674843527 0.4050331651689163

0.7500020258638074 0.5026996617448631 0.4053481233041012

0.2543214409763834 0.0070652513611407 0.4056130065973775

0.0025299380062478 0.5035714993758518 0.4066807380842761

0.0026238622588153 0.7543921180551507 0.4066936854502714

0.2534225355080366 0.7544917606731403 0.4067804826281147

0.0037056280978263 0.2541536044592618 0.4070231860074074

0.7520237285292026 0.7533336582199226 0.4070807719520460

0.5029316676500315 0.7533617973806486 0.4070812932429178

0.7519607421689898 0.2542060229862386 0.4071634251873135

0.0037732212496451 0.0050297033555466 0.4071873411857396

0.5028044959786300 0.0051120761115939 0.4073177231400678

0.7528427193524649 0.0041285777070712 0.4078146135187097

0.4194959864634953 0.3374465438360446 0.3335811777659714

0.4219038178906658 0.5881837871172308 0.3350079192144477

0.1689756944234801 0.3351297907094590 0.3350216611195407

0.6680398553678586 0.5880676889532452 0.3351139086359112

0.6677248199621388 0.3352373532014212 0.3352336358027017

0.1691131290410510	0.0890696977718122	0.3353298881526001
0.4215842164624988	0.0891125086516240	0.3353885991656824
0.1695117820167482	0.5876456897667808	0.3362878440880124
0.9194397932760126	0.5874150105549774	0.3363229445210365
0.1697524725691011	0.8377845746588974	0.3364644685448752
0.9189443639168786	0.3365278421239069	0.3368749950854308
0.9191703846416104	0.8380377008217863	0.3368778199910807
0.4204805859122231	0.8381841984905307	0.3370244377007978
0.6697835326779753	0.8379774422480253	0.3372919464467046
0.9190965828691446	0.0871600138536266	0.3373326706395119
0.6695448972739513	0.0872895554805027	0.3374514240201592
0.4189052540122304	0.5810711324766302	0.4754069923966853
0.1758189816545439	0.3382030499823827	0.4754788768297686
0.6638731055497971	0.5813741627254529	0.4756991182939601
0.6637865178141595	0.3380893583195004	0.4758896096482559
0.1757796288457101	0.0934471855870171	0.4759599794279978
0.4190727217371659	0.0935193642548100	0.4760640598052035
0.1707407467996234	0.5861777386531557	0.4761370382289840
0.9173074593137542	0.5863537432409345	0.4761863660960941
0.1704890399469434	0.8395350109241519	0.4763280412292943
0.9197374968398933	0.3377500647306704	0.4773274298071684
0.9196226489667580	0.8372583035310228	0.4773293233755069
0.4192690678646471	0.8372257381627648	0.4774565425488362
0.9206186176947071	0.0880846197914238	0.4778034767083952
0.6690128083297040	0.8363713503638621	0.4778230420106482
0.6691112340973646	0.0880960914526909	0.4778595129325183
0.4490265894829548	0.3618201484405361	0.5836993772570125
0.5111376731774584	0.5897063919761365	0.6159225819632610
0.2164658423109955	0.2798043379403055	0.6169233633045152
0.2708920005584829	0.5120565018161258	0.6255680252508355
0.4486632625562947	0.3445746691217693	0.5407567373776487
0.2457819311178469	0.5479490417682413	0.5938718973808790
0.3612227056721977	0.3206880025567784	0.5957393845524663
0.4993475498273696	0.5086070672241263	0.6031270166664697
0.2254251958363511	0.3626929698206567	0.6209864992445162
0.3615923652796553	0.5525084168715821	0.6227391294131976
0.5601278653520438	0.6113139536197352	0.6524204334173831

0.1904728554038329 0.2420392419553181 0.6563916434235324

(j) Optimized geometry for $(H_2O)_5$ on defective MoS_2 monolayer:

Mo S O H

1.00000000000000

12.7335259999999995 0.0000000000000000 0.0000000000000000

-6.3667629999999997 11.0275569957496469 0.0000000000000000

0.0000000000000000 0.0000000000000000 22.2171659999999989

Mo S O H

16 31 5 10

Direct

0.5117900553082251 0.3568287974309499 0.4040100760215495

0.2683697884966207 0.3570726598488960 0.4040121452817544

0.5118416801035934 0.6003825442439137 0.4040393163410982

0.2655769134497135 0.6034047749537876 0.4051494327171952

0.2655036356003606 0.1076171006671132 0.4052685599757027

0.7613434317658800 0.6034079042560805 0.4053548014385129

0.0139635444994752 0.6042865580753585 0.4063682672841153

0.0140729821192593 0.8550493810103390 0.4064163783156829

0.2646446332818400 0.8549788934034623 0.4065695554075361

0.7633023400182637 0.3547477651383488 0.4068531208966490

0.5140594098076292 0.8537971828693856 0.4068550186860804

0.0150219840780466 0.3547851540131077 0.4068942552609016

0.0151011656095363 0.1057770138435927 0.4069090096356476

0.7632388370366385 0.8538725723204337 0.4069897221264611

0.5139251120308970 0.1055100472041133 0.4070575589197958

0.7641812398680301 0.1046946792797740 0.4075373213375002

0.4306830345566510 0.4380435497763315 0.3334905552423209

0.4329264590208481 0.6884415289585988 0.3349788638602007

0.1803555446785339 0.4359792818580033 0.3350028327342613

0.1804200878685478 0.1898817951377296 0.3350251563430930

0.4328051157423971 0.1896785402425039 0.3351028607737661

0.6790048046078425 0.4359651096291017 0.3351104014135728

0.6791110592302374 0.6885078564390099 0.3351920151571619

0.1809923450620801 0.6882232236509225 0.3360622390367141

0.9305866651197263 0.6880187727327822 0.3361185965953624

0.1810921273167025 0.9383365121553382 0.3361619044221271

0.9303122314216452	0.4371099171675610	0.3365705738788876
0.9303748722307859	0.9386444603019584	0.3367095877625985
0.4316675679347597	0.9385872455153219	0.3367434508112339
0.6810105215425679	0.9383996319673145	0.3370703311156760
0.9304760637777392	0.1878871708267766	0.3370752187429176
0.6807430543079462	0.1877202501615045	0.3371768480419846
0.1871677533301934	0.1941438640912452	0.4755077718615439
0.1870267099630993	0.4388019914993038	0.4756091521268360
0.6750978593532935	0.4384555220255493	0.4756386732519573
0.4300822775964477	0.1939408613577740	0.4756430021197176
0.4301961142584503	0.6818498340311905	0.4757073842767880
0.6748849563175341	0.6818412636778106	0.4757596547038716
0.9290046937945178	0.6871180678983606	0.4759900103735717
0.1817717532707519	0.6868581300211858	0.4760437170737148
0.1818333621251540	0.9401310770026186	0.4761052266539565
0.9310027127855918	0.4385132544937704	0.4770303815586345
0.9312018364318462	0.9380408550700210	0.4771207948287000
0.4304958419919274	0.9376769896929531	0.4772297598387709
0.6801114679450393	0.9369832985003725	0.4775433567438014
0.6803851675261257	0.1886242263088178	0.4775807338916280
0.9318952449497715	0.1886739876875865	0.4775982164567267
0.4276652981249782	0.4393010739846019	0.5812861803926168
0.1970524601730830	0.3088694548218669	0.6167108839321287
0.3573101491355288	0.0804144285990702	0.6239703260542555
0.5302219026223227	0.3150755061593387	0.6248442200700808
0.1474321545467205	0.0793821362879470	0.6252705104848530
0.4351088675903938	0.4431942811808582	0.5373770202181092
0.4711774679024003	0.3957888145098352	0.5952288207023884
0.0839168643093231	0.0323399412837304	0.5960105766487516
0.2846226331506401	0.3611957838647655	0.6040641974071193
0.6025258905090070	0.3230959934211910	0.6054076030138731
0.1623902559385115	0.1648862055175755	0.6225256600154836
0.2760630884477990	0.0763520792195536	0.6230347005281018
0.4688766068125929	0.2256655374959209	0.6247460162125975
0.1882575519117609	0.3456139301028003	0.6534528138152425
0.3556564169295982	0.0370478788734161	0.6604476704953157
